# Determination of caffeine in treated wastewater discharged in the Nile River with emphasis on the effect of zinc and physicochemical factors

**DOI:** 10.1007/s11356-024-32918-6

**Published:** 2024-03-26

**Authors:** Nouran A. I. Tawfik, Zienab A. El-Bakary, Khaleid F. Abd El-Wakeil

**Affiliations:** Zoology and Entomology Department, Faculty of Science, Assiut University, Assiut, Egypt

**Keywords:** Emerging contaminants, Pharmaceuticals, Micropollutants, Caffeine, Zinc, Water pollution, Water quality

## Abstract

The present study aimed to investigate the occurrence of caffeine residues in the Nile River according to drainage of treated wastewater at Assiut, Egypt, and the effects of physicochemical parameters and zinc on its concentration. Four different sites were selected to perform the study: S, wastewater treatment plant (WWTP) canal (source site); J, a junction site between WWTP canal and the Nile; R, a reference site in the Nile before J site; and A, a site located after J site in the Nile. Water and sediment samples were collected in Summer 2022 and Winter 2023. Caffeine and Zn concentrations and physicochemical parameters were measured in the collected samples. The caffeine concentrations in water samples ranged from 5.73 to 53.85 μg L^−1^ at S in winter and summer, respectively, while those in sediment ranged from 0.14 mg kg^−1^ at R in winter to 1.54 mg kg^−1^ at S in summer. Caffeine and Zn concentrations were higher in summer samples. The Water Quality Index (WQI) of the collected samples recorded the lowest values in winter season at S and J sites. The study found that caffeine and zinc concentrations are positively correlated with water temperature and conductivity, while negatively correlated with pH. The association between caffeine and Zn highlights the environmental impact of heavy metals and pharmaceutical residues, and stresses the need for future research on these interactions.

**Supplementary Information:**

The online version contains supplementary material available at 10.1007/s11356-024-32918-6.

## Introduction

Many chemicals and compounds that are not controlled yet are becoming increasingly harmful to the quality of freshwater ecosystems, such as pharmaceuticals (PhACs) and personal care products (PCPs) (Ortúzar et al. 2022). Given the growing use of PhACs in human and animal medicine (Ebele et al. 2020), active chemical residues in ocean, surface water, groundwater and even drinking water are becoming more common (Ebele et al. 2020; Hawash et al. 2023). Recently, the continuous discharge of pharmaceutical and personal care products (PPCPs) in natural waters have received a lot of interest (Korekar et al. 2020; Mohammad and Abd El-wakeil 2021) and it was comprehensively discovered in the worldwide environment (Hawash et al. 2023).

Pharmaceutical residues are chemical composites that contaminate waterways and soil, producing severe difficulties for environmental health and non-target organisms (Mohammad et al. 2021). Furthermore, through food chains, some plants can carry these chemicals from the soil to animals and humans (Tiwari et al. 2017; Mohammad et al. 2021). Water pollution by PhACs has been a concern since the 1990s (Doerr-MacEwen and Haight 2006), with findings revealing the presence of pharmaceutical chemicals and their metabolites in the ng L^−1^ to mg L^−1^ range in freshwater environments. PhACs pass in the aquatic environment via public effluents, and detoxification methods in wastewater treatment plants (WWTPs) appear insufficient to control them. The consistent outflow immediately affects the aquatic habitat, giving it pseudo constancy (Hernando et al. 2006).

Caffeine is a central nervous system stimulant (Asghar et al. 2018). As a common ingredient of caffeinated food and beverages (e.g. chocolate, coffee, cocoa, tea, dairy desserts and soft drinks), caffeine is the most extensively used medication in the world, accounting for cold remedies, analgesics, stimulants and illegal narcotics (Kosma et al. 2014), as well as one of the most representative pharmaceutical residue pollutants detected in the water environment (Pires et al. 2016). Concentrations (ng L^−1^) of caffeine were detected in surface water in various regions of the world as well as in tap/drinking water (Hawash et al. 2023). Lv et al. (2019) detected high levels of caffeine (607.85 ng L^−1^) in drinking water in China. However, it was discovered that locations out of human impact, like Antarctica, are affected by caffeine pollution (Li et al. 2020a). Abdallah et al. (2019) determined the presence of 30 PPCPs, including caffeine, in the effluent of five WWTPs and five surface water samples collected (7 to 54 ng L^−1^) in Egypt’s Assiut Governorate. Caffeine concentrations in the environment vary from 2 to 1600 ng L^−1^, with higher levels recorded for estuary and coastal waters (Korekar et al. 2020; Li et al. 2020b). Li et al. (2020b) referred to caffeine as an indicator for anthropogenic pollution as well as a source-specific indicator for wastewater in surface waters.

Despite the fact that caffeine has outstanding removal efficiency during wastewater treatment, the amount of caffeine that enters the water is substantially greater than that which is degraded (Zhu et al. 2013). Furthermore, caffeine residue in the aquatic environment is exceptionally stable, with a reported half-life of 100–240 days (Hillebrand et al. 2012). Caffeine is thus classed as an emerging pollutant, a drug that is not currently monitored (USEPA 2016), but should be included in future laws because it is commonly found and can impair aquatic biota like algae, plankton, benthos and fish (Stuart et al. 2012). Caffeine’s continual presence in aquatic habitats creates interest in studying its occurrence, concentration and bioaccumulation in freshwater ecosystem.

Heavy metals are severe pollutants in the environment due to their toxicity, persistence, pervasive nature and non-biodegradability (Pekey 2006; Wu et al. 2016) and have a variety of adverse sub-lethal and deadly impacts on aquatic organisms (Peters et al. 1997). Additionally, heavy metal pollution can have a disastrous impact on the recipient environment’s biological balance and a variety of aquatic creatures (Vosyliene and Jankaite 2006). Heavy metals pose a threat due to their inability to biodegrade and their ability to accumulate in soil, where they are transferred to organisms that live in water via nutrition or respiration, and then bioaccumulation of higher-level organisms (Gedik and Boran 2013). Heavy metal pollution is primarily caused by human activities like mining, smelting and foundries, as well as leaching from sources like landfills, waste dumps and roadworks. Secondary sources include agricultural use and natural factors like volcanic activity and erosion (Briffa et al. 2020).

One of the most important heavy metals is zinc (Zn), which is the second most abundant trace metal in the human body after iron. Zn is an essential trace element that plays an important role in the growth and development of animals and humans (Read et al. 2019). Although Zn is necessary for life, it is hazardous in large amounts (Taylor et al. 1982) and, through bioaccumulation processes, might harm creatures at higher trophic levels in the food chain, leading to significant impacts on human life and ecosystems (Ip et al. 2007). Zhou et al. (2016a) showed that bioaccumulation of Zn in the human body caused by a heavy metal–contaminated diet can impair the immune system and disrupt the high-density lipoprotein. In the pharmaceutical sector, zinc compounds are utilised as components in commodities such as vitamin supplements, diaper rash ointments, sunblock, deodorants and antidandruff shampoos (McComb et al. 2014). As the whole world is grappling with coronavirus disease 2019 (COVID-19) pandemic and the lack of clinically effective therapies, attention is shifting to different ways of strengthening the immune system. Zn has produced a lot of excitement as one of the promising applicants to reduce the harshness of COVID-19 infection. Zn, a well-known anti-inflammatory and antioxidant mineral contained in diet, is currently being employed in numerous studies against COVID-19 (Pal et al. 2021). Therefore, world’s zinc production is still increasing. This essentially means that more zinc enters to environment. Zn concentrations in many freshwaters are increased by domestic and industrial waste or other sources (Taylor et al. 1982). Zn is considered as one of the utmost extensively known pollutants in the aquatic ecosystems (Brinkman and Johnston 2012).

WWTPs are considered a main input path for micropollutants into aquatic environments (Korekar et al. 2020; Li et al. 2020b). The primary objective of the present study was to investigate the occurrence and concentrations of caffeine residues and Zn in the Nile River according to drainage of WWTPs at Assiut Province, Egypt. Also, the study aimed to assess some physicochemical parameters (air/water temperature, pH, electric conductivity, total dissolved salts, turbidity, dissolved oxygen, organic matter, phosphorus, nitrate and ammonia) and their interaction on caffeine and Zn concentrations during extreme summer and winter seasons. Additionally, the studied physicochemical parameters were used to assess and calculate Water Quality Index (WQI) at investigated sites during studied seasons.

## Materials and methods

### Sampling

**Fig. 1:**
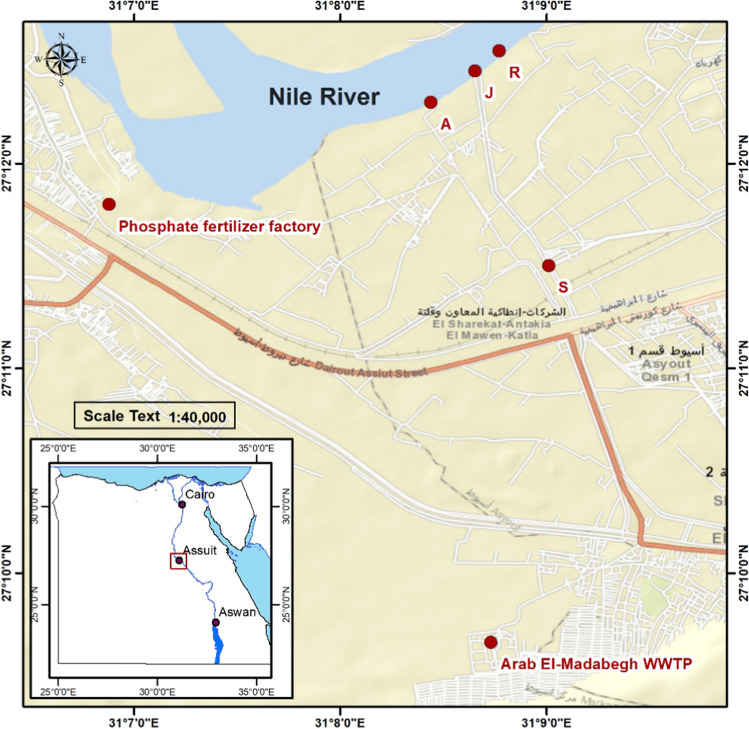
Maps showing the study sites at Assiut Governorate, Egypt. S, source-of-pollution site, where treated water is discharged from wastewater treatment plant effluent (Arab El-Madabegh WWTP); J, junction site where the water of the wastewater treatment canal meets with water flow of the Nile; R, reference site in the Nile River before the junction site; A, site located after the junction point in the Nile River

The collection of water and sediment samples was carried out on the 31st of July 2022 (summer) and 2nd of February 2023 (winter) from Assiut Province, Egypt (27° 14′ N, 31° 11′ E). Samples were collected in triplicates. Sediment samples were stored in plastic cases, while water samples were kept in 1.5-L polyethylene bottles. The samples were kept cold by being placed in an icebox filled with ice prior to reaching the laboratory for analysis. Four different sites were selected to perform the present study (Fig. 1): site 1 (S), a treated wastewater canal (source of pollution) where treated water is discharged from wastewater treatment plant effluent (Arab El-Madabegh WWTP); site 2 (J), a junction site where the water of the wastewater treatment canal meets with water flow of the Nile; site 3 (R), a reference site in the Nile River before the junction site; and site 4 (A), a site located after the junction point in the Nile River.

### Physicochemical parameters

Physicochemical investigations of samples were carried out in accordance with the protocols outlined in the American Public Health Association (APHA 2017). During sampling, air temperature/water temperature (AT/WT, °C), water pH and electrical conductivity (Cond, μS cm^−1^) were in situ using an Eutech instrument (EcoScan pH 6). A total dissolved solid (TDS, ppm) was measured by a digital TDS handheld meter (hold). Transparency (turbidity (Turb), cm) was assessed by a white/black Secchi disc (20 cm in diameter). Dissolved oxygen (DO, mg L^−1^) was determined by using a Mic portable water quality meter (model 98725). In Central Laboratory for Chemical Analysis, Faculty of Agriculture, Assiut University, sediment organic matter (OM, %) was determined according to Ben-Dor and Banin (1989). Water phosphate (PO_4_, mg L^−1^), nitrate (NO_3_, mg L^−1^) and ammonia (NH_4_, mg L^−1^) were determined using a spectrophotometer according to APHA-AWWA-WPCF (1989). Hanna Instruments set kits of PO_4_, NO_3_ and NH_4_ were used to perform the analyses. Concentrations of Zn in water and sediment samples were determined by using the inductively coupled plasma emission spectrometer (iCAP 6200) according to Jackson (1974). The wavelength used for the detection and measurement of Zn was 213.856 nm. The limit of quantification (LOQ) was 0.0034 ppm with 98.78% confidence level. The precision of the Zn concentration was validated by repeating every sample three times.

### Determination of caffeine concentration

Caffeine concentrations in sediment and water samples were determined in Multidisciplinary Research Center of Excellence, Assiut University (MIRCE), by means of a single flow-through UV multiparameter sensor (a multiparameter-responding flow-through system with solid-phase UV spectrophotometric detection (a multiparameter optosensor)) (Vidal et al. 2003). The accuracy of caffeine detection was calculated with triple measurements. The linearity of the calibration range was between 0.2 and 2 ppm. The concentration reached the detection zone and was measured at 273 nm. The LOQ was 0.1 ppm with 99.9% confidence level.

### Determination of WQI

WQI used refers to the CCMEWQI method (CCME 2001) and was settled to estimate the overall water quality status of investigated samples. Ten water quality parameters were used. These parameters are temperature (°C), pH, conductivity (μS cm^−1^), dissolved oxygen (mg L^−1^), total dissolved solids (mg L^−1^), nitrate (mg L^−1^), ammonium (mg L^−1^), phosphate (mg L^−1^), turbidity (nephelometric turbidity units (NTU)) and Zn concentration (mg L^−1^). It is worth mentioning that the turbidity Secchi disc transparency measurements were converted into standard NTU according to Baughman et al. (2015). The minimum Egyptian standards for the water quality of the Nile River according to Law 48/1982 (EEAA 1999) were used for WQI calculation. There are five classes of the WQI to describe water quality as excellent, good, fair, marginal and poor when the value of the WQI lies between 95 and 100, between 80 and 94, between 65 and 79, between 45 and 64, and between 0 and 44, respectively.

### Statistical analysis

Excel Office 2013, IBM SPSS Statistics (version 20) and PAST4 program performed data summary and analysis. Two-way analysis of variance (ANOVA) was applied to investigate for significant differences of physicochemical parameters between the investigated sites and seasons followed by the Duncan test to determine pairwise differences between means. Pearson correlation and stepwise multiple regression were used to consider association between the investigated physicochemical parameters and caffeine and Zn concentrations. The PAST4 program was used to perform the distance-based two-way permutational multivariate analysis of variance (PERMANOVA) to investigate the influence of physicochemical parameters on collected samples followed by PERMANOVA pairwise tests to verify the significance of the differences among samples. After standardizing the collected data, a hierarchical cluster and principal component analyses (PCA) of the mean values of investigated physicochemical parameters and caffeine and Zn concentrations were applied.

## Results

### Physicochemical parameters

**Fig. 2:**
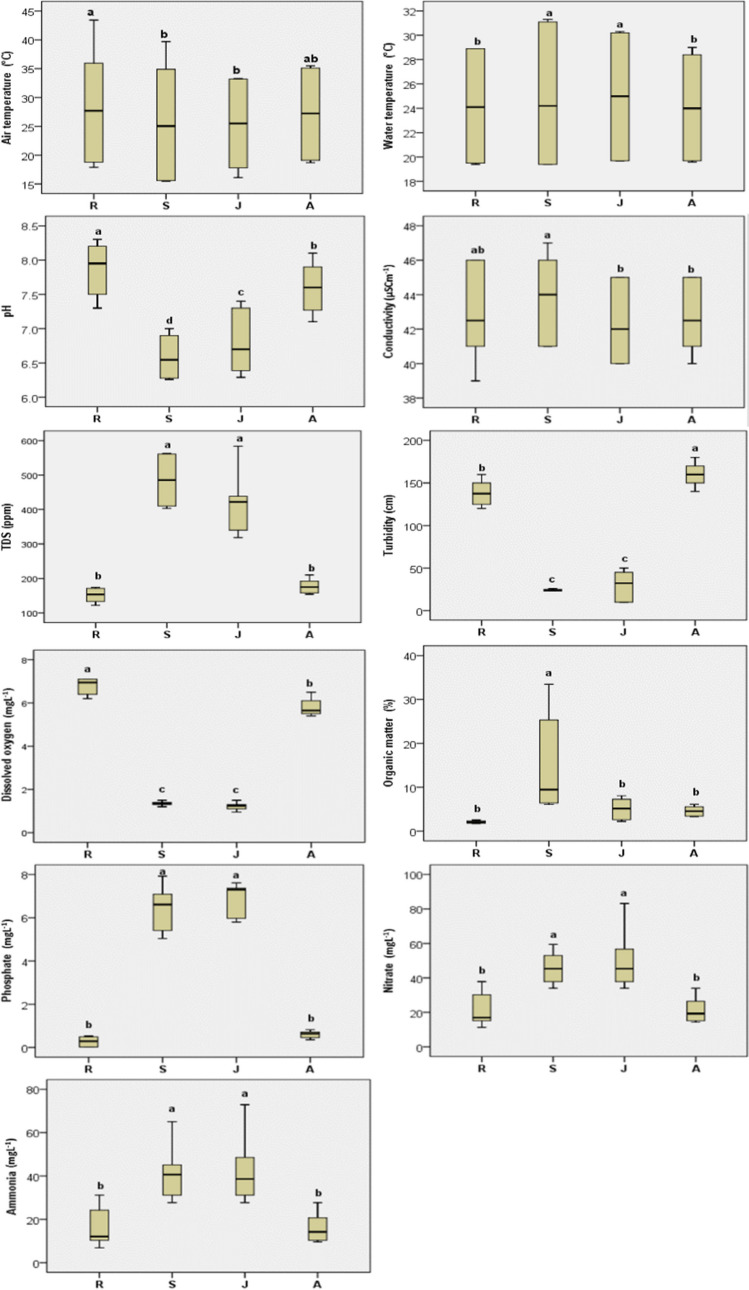
Boxplots of investigated physicochemical parameters at study sites with statistical results (similar letters for each variable show no significant difference at the 0.05 level)

Table 1 shows the summarized results of physicochemical parameters at the study sites during the investigated seasons. Statistical result showed that the differences among sites were significant for all investigated parameters (*p* < 0.05), whereas the differences between seasons were non-significant in the case of transparency and dissolved oxygen and significant for the rest of parameters (Supplementary material 1). The mean temperature of the collected water samples at the four sites ranged from 19.43 °C in winter to 30.43 °C in summer, while air temperature changes ranged from 15.57 °C in winter to 38.29 °C in summer. Water temperatures at S and J sites showed statistically significant higher values than those at R and A sites (Fig. 2).

**Table 1:** Mean (*M*) ± standard deviation (SD) of physicochemical parameters for the study sites during the investigated seasons

Physicochemical parameters	R		S		J		A	
	Sum	Win	Sum	Win	Sum	Win	Sum	Win
	*M* ± SD	*M* ± SD	*M* ± SD	*M* ± SD	*M* ± SD	*M* ± SD	*M* ± SD	*M* ± SD
AT (°C)	38.29 ± 4.45	18.87 ± 1.00^a^	36.37 ± 2.89	15.57 ± 0.06^a^	33.20 ± 0.10	17.27 ± 1.01^a^	35.00 ± 0.56	19.30 ± 0.72^a^
WT (°C)	28.83 ± 0.12	19.47 ± 0.06^a^	30.43 ± 1.33	19.43 ± 0.06^a^	30.20 ± 0.10	19.77 ± 0.12^a^	28.57 ± 0.38	19.67 ± 0.06^a^
pH	7.50 ± 0.20	8.23 ± 0.06^a^	6.28 ± 0.02	6.90 ± 0.10^a^	6.39 ± 0.11	7.20 ± 0.26^a^	7.22 ± 0.11	7.97 ± 0.12^a^
Cond (μS cm^−1^)	45.33 ± 1.15	40.33 ± 1.15^a^	46.00 ± 1.00	41.67 ± 1.15^a^	44.67 ± 0.58	40.00 ± 0.00^a^	44.67 ± 0.58	40.67 ± 0.58^a^
TDS (ppm)	131.33 ± 8.62	171.00 ± 3.00^a^	408.33 ± 4.73	561.00 ± 2.00^a^	427.33 ± 9.71	414.33 ± 147.31	157.33 ± 3.06	197.33 ± 11.02^a^
Turb (cm)	126.67 ± 7.64	150.00 ± 10.00	24.00 ± 1.00	24.33 ± 1.53	45.00 ± 5.00	15.00 ± 8.66^a^	150.00 ± 10.00	170.00 ± 10.00^a^
DO (mg L^−1^)	6.87 ± 0.40	6.70 ± 0.44	1.37 ± 0.15	1.33 ± 0.06	1.08 ± 0.13	1.37 ± 0.12^a^	5.53 ± 0.15	6.07 ± 0.45
OM (%)	1.94 ± 0.13	2.17 ± 0.42	6.48 ± 0.48	23.52 ± 10.87^a^	7.18 ± 0.94	2.96 ± 1.00^a^	4.09 ± 1.23	5.05 ± 1.28
PO_4_ (mg L^−1^)	0.45 ± 0.11	0.10 ± 0.13^a^	7.28 ± 0.57	5.61 ± 0.70^a^	7.43 ± 0.16	6.35 ± 0.82	0.71 ± 0.12	0.50 ± 0.17
NO_3_ (mg L^−1^)	13.86 ± 2.18	28.98 ± 9.51^a^	37.80 ± 3.78	53.81 ± 5.18^a^	37.80 ± 3.78	63.00 ± 17.86	15.16 ± 0.84	27.72 ± 5.77^a^
NH_4_ (mg L^−1^)	9.15 ± 2.01	23.06 ± 8.75^a^	32.84 ± 6.15	50.58 ± 12.64	31.51 ± 3.99	544.36 ± 16.44	10.35 ± 0.78	21.90 ± 5.31^a^

*AT* air temperature, *WT* water temperature, *pH* water pH, *Cond* conductivity, *TDS* total dissolved solids, *Turb* turbidity, *DO* dissolved oxygen, *OM* organic matter, *PO*_*4*_ phosphate, *NO*_*3*_ nitrate, *NH*_*4*_ ammonia, *Sum* summer, *Win* winter

^a^Significant differences between seasons at the 0.05 level

The pH of water samples in the study ranged from 6.28 to 8.23 in summer at S site and in winter at R site, respectively (Table 1). Water pH was in the alkaline range at R and A sites while it was slightly acidic in S and J sites (Fig. 2). The mean EC of the analysed water samples at the four sites fluctuated between 40 and 46 μS cm^−1^. The TDS concentration of the collected water samples was within the range between 131.33 and 561.00 ppm (Table 1). The S site recorded the highest values for both conductivity and TDS followed by the J site (Fig. 2). DO concentrations showed variable results according to site and season. In summer, DO ranged from 1.08 to 6.87 mg L^−1^ at J and R sites, respectively. In winter, DO ranged from 1.33 to 6.70 mg L^−1^ at S and R sites, respectively (Table 1). DO concentrations were significantly higher at R and A sites than at S and J sites (Fig. 2).

The highest sediment OM value was 23.52 in winter at S site, and the lowest was 1.94 in summer at R site (Table 1). OM was significantly higher at S site than at J, A and R sites (Fig. 2). The concentration levels of PO_4_ of the collected water samples at four sites ranged from 0.10 to 7.43 mg L^−1^, while the level of NO_3_ ranged between 13.86 and 63.00 mg L^−1^. NH_4_ concentrations showed variable results according to site and season. In summer, NH_4_ ranged from 9.15 to 32.84 mg L^−1^ at R and S sites, respectively. In winter, NH_4_ ranged from 21.90 to 54.36 mg L^−1^ at A and J sites, respectively (Table 1). PO_4_, NO_3_ and NH_4_ levels were significantly higher at S and J sites than at A and R sites (Fig. 2).

### Caffeine and Zn concentrations in water and sediment

The present results of caffeine and Zn concentrations in water and sediment for the study sites during the investigated seasons are represented in Table 2. Both caffeine and zinc showed fluctuations among study samples. Statistical results showed that these variations were significant among sites and seasons in case of water and sediment caffeine and sediment Zn while the variation of Zn in water was non-significant (Supplementary material 2).

**Table 2:** Mean (*M*) ± standard deviation (SD) of caffeine and Zn concentrations in water and sediment for the study sites during the investigated seasons

Sites	Season	WCaf (μg L^−1^)	SCaf (mg kg^−1^)	WZn (mg L^−1^)	SZn (mg kg^−1^)
		*M* ± SD	*M* ± SD	*M* ± SD	*M* ± SD
R	Sum	23.72 ± 4.27	0.88 ± 0.13	0.17 ± 0.05	77.85 ± 17.42
	Win	7.13 ± 0.95^a^	0.14 ± 0.01^a^	0.16 ± 0.11	60.62 ± 8.86
S	Sum	53.85 ± 11.54	1.54 ± 0.33	0.18 ± 0.03	139.05 ± 22.25
	Win	5.73 ± 0.55^a^	0.23 ± 0.03^a^	0.08 ± 0.02^a^	124.37 ± 13.47
J	Sum	45.51 ± 6.75	0.88 ± 0.33	0.17 ± 0.01	155.02 ± 42.78
	Win	8.13 ± 2.57^a^	0.20 ± 0.07^a^	0.08 ± 0.04^a^	28.59 ± 28.48^a^
A	Sum	40.32 ± 4.05	1.02 ± 0.14	0.15 ± 0.05	146.37 ± 63.66
	Win	8.13 ± 0.95^a^	0.18 ± 0.04^a^	0.22 ± 0.04	99.89 ± 22.32

*WCaf* water caffeine, *SCaf* sediment caffeine, *WZn* water zinc, *SZn* sediment zinc, *Sum* summer, *Win* winter

^a^Significant differences between seasons at the 0.05 level

**Fig. 3:**
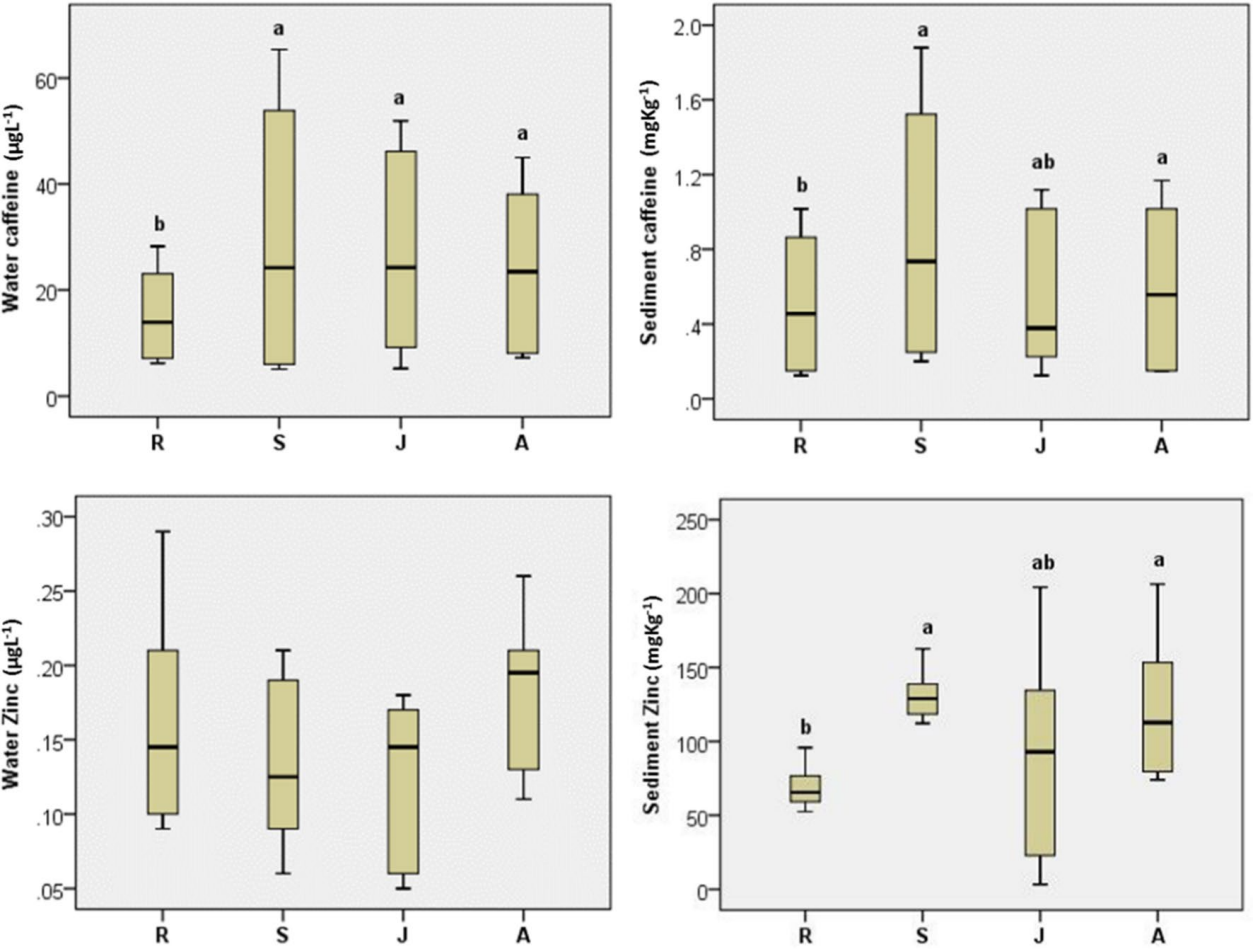
Boxplots of caffeine and Zn concentrations in water and sediment at study sites with statistical results (similar letters for each variable show no significant difference at the 0.05 level)

The variations in water caffeine (WCaf) ranged between 23.72 μg L^−1^ at R site and 53.85 μg L^−1^ at S site in summer, while in winter, the maximum value was recorded at both J and A sites (8.13 μg L^−1^), and the minimum value was recorded at S site (5.73 μg L^−1^). The values of sediment caffeine (SCaf) ranged from 0.14 mg kg^−1^ at R site during winter to 1.54 mg kg^−1^ at S site during summer (Table 2). S site caffeine in both water and sediment showed significantly higher concentrations, in contrast to R site which showed the lowest concentration (Fig. 3).

The mean water zinc (WZn) of studied sites ranged from 0.08 mg L^−1^ at S and J sites in winter to 0.22 mg L^−1^ at A site in winter, while sediment zinc concentrations ranged between 28.59 mg kg^−1^ at J site in winter and 155.02 mg kg^−1^ at J site in summer (Table 2). The differences in WZn concentrations were statistically non-significant, while sediment zinc (SZn) concentrations at S, J and A sites was significantly higher than that at R site (Fig. 3).

### The extent of differences between collected samples

**Fig. 4:**
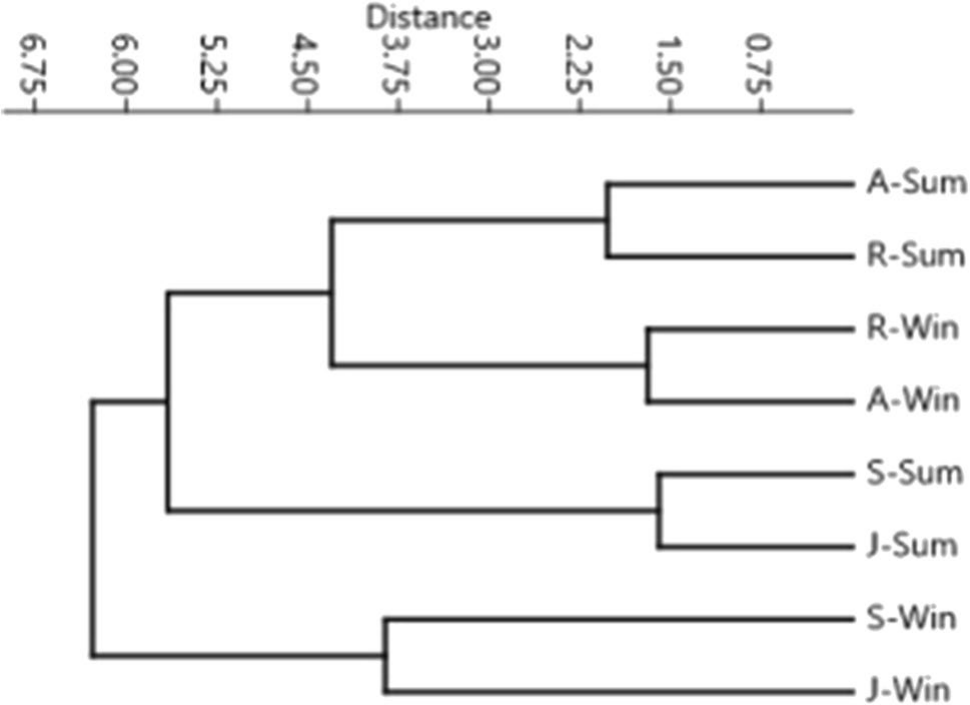
Dendrogram shows the collected samples’ classification at the investigated sites during the summer and winter seasons based on the physicochemical parameters and caffeine and Zn concentrations in water and sediment (after standardizing the collected data)

Two-way PERMANOVA for investigated physicochemical parameters and caffeine and Zn concentrations in water and sediment of the studied sites and seasons indicated that there are significant differences among sites (*F* = 21.815, *p* = 0.0001) seasons (*F* = 54.107, *p* = 0.0001) and interactions (*F* = 3.638, *p* = 0.0012) (Table 3). Pairwise tests indicated that S and J sites differ significantly from R and A sites; however, there were no significant differences between S and J sites and between R and A sites (Table 4). At a Euclidean distance of 5.5, the results classified the collected samples into three clusters. The first cluster consisted of winter samples from S and J sites. The second cluster consisted of summer samples from S and J sites. The third cluster consisted of samples from A and R sites in both winter and summer seasons (Fig. 4).

**Table 3:** Two-way PERMANOVA for physicochemical parameters and caffeine and Zn concentrations in water and sediment at the investigated sites during summer and winter seasons

Source	Sum of squares	*df*	Mean square	*F*	*p* value
Sites	154.15	3	51.384	21.815	0.0001
Season	127.45	1	127.45	54.107	0.0001
Interaction	25.711	3	8.5703	3.638	0.0012
Residual	37.688	16	2.3555		
Total	345	23

**Table 4:** PERMANOVA pairwise tests for differences between the investigated sites according to standardized physicochemical parameters and caffeine and Zn concentrations during the period of study

Sites	*F*	*p* value
R-S	8.635	0.002
R-J	7.554	0.0027
R-A	0.8722	0.4999
S-J	0.8933	0.4799
S-A	7.231	0.0034
J-A	7.031	0.0043

A, a site located after the junction site in the Nile; J, a junction site between WWTP canal and the Nile; R, a reference site in the Nile before the junction site; S, wastewater treatment plant canal (source site)

### Water Quality Index

**Fig. 5:**
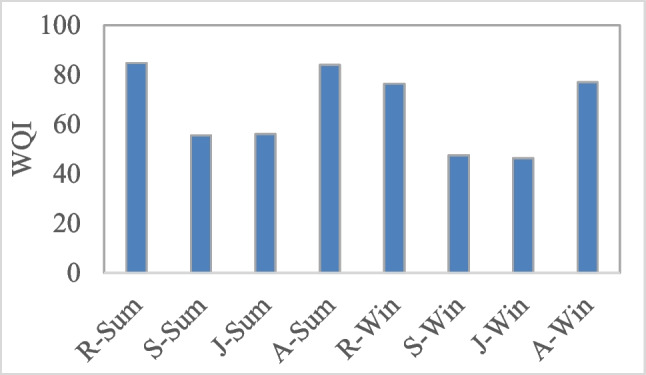
Water Quality Index (WQI) for the collected samples of the study sites during the investigated seasons

Figure 5 shows the WQI for the collected samples. The quality of water for the collected samples ranged from good to marginal quality. Samples collected from R and A sites during summer had a good quality (WQI = 83.9% and 84.7%, respectively). Winter samples from R (76.3%) and A (76.9%) sites had a fair quality. On the other hand, the quality of water samples from S (47.5% and 55.4%) and J (46.3% and 56.1%) sites (during winter and summer seasons, respectively) was marginal quality.

### Effect of physicochemical parameters on caffeine and Zn concentrations

The correlations between the studied physicochemical parameters and caffeine and Zn concentrations in water and sediment are illustrated in Table 5. WCaf had a strong negative correlation with pH (*r* = − 0.688) and a strong positive correlation with AT, WT and Cond (*r* = 0.804, *r* = 0.898 and *r* = 0.857, respectively). SCaf had a strong positive correlation with AT, WT, Cond and WCaf (*r* = 0.853, *r* = 0.879, *r* = 0.869 and *r* = 0.885, respectively), whereas SCaf had a strong negative correlation with pH (*r* = − 0.0623). WZn showed a negative correlation with NO_3_ and NH_4_ (*r* = − 0.439 and *r* = − 0.430, respectively). SZn presented a weak negative relation with pH (*r* = − 0.476) and a positive relation with AT, WT, Cond, WCaf and SCaf (*r* = 0.433, *r* = 0.526, *r* = 0.513, *r* = 0.548 and *r* = 0.548, respectively).

**Table 5:** Pearson correlation coefficients (*r*) between the investigated physicochemical parameters and caffeine and Zn concentrations in study sites

	AT	WT	pH	Cond	TDS	Turb	DO	OM	PO_4_	NO_3_	NH_4_	WCaf	SCaf	WZn
WT	0.957^a^													
pH	− 0.435^b^	− 0.612^a^												
Cond	0.873^a^	0.913^a^	− 0.591^a^											
TDS	− 0.304	− 0.102	− 0.695^a^	− 0.066										
Turb	0.076	− 0.098	0.770^a^	− 0.094	− 0.879^a^									
DO	0.107	− 0.110	0.808^a^	− 0.075	− 0.900^a^	0.939^a^								
OM	− 0.331	− 0.226	− 0.309	− 0.132	0.931^a^	− 0.430^b^	− 0.462^b^							
PO_4_	0.003	0.218	− 0.852^a^	0.173	0.877^a^	− 0.942^a^	− 0.970^a^	0.380						
NO_3_	− 0.574^a^	− 0.434^b^	− 0.301	− 0.438^b^	0.705^a^	− 0.744^a^	− 0.752^a^	0.366	0.672^a^					
NH_4_	− 0.564^a^	− 0.421^b^	− 0.323	− 0.390	0.723	− 0.742^a^	− 0.749^a^	0.365	0.666^a^	0.987^a^				
WCaf	0.804^a^	0.898^a^	− 0.688^a^	0.857^a^	0.041	− 0.192	− 0.272	− 0.159	0.363	− 0.289	− 0.281			
SCaf	0.853^a^	0.879^a^	− 0.623^a^	0.869^a^	− 0.031	− 0.184	− 0.178	− 0.148	0.260	− 0.332	− 0.322	0.885^a^		
WZn	0.289	0.254	0.082	0.264	− 0.333	0.394	0.359	− 0.320	− 0.243	− 0.439^b^	− 0.430^b^	0.263	0.257	
SZn	0.433^b^	0.526^a^	− 0.476^b^	0.513^b^	0.143	− 0.084	− 0.241	0.335	0.210	− 0.179	− 0.152	0.548^a^	0.548^a^	0.198

^a^Correlation is significant at the 0.01 level

^b^Correlation is significant at the 0.05 level

**Fig. 6:**
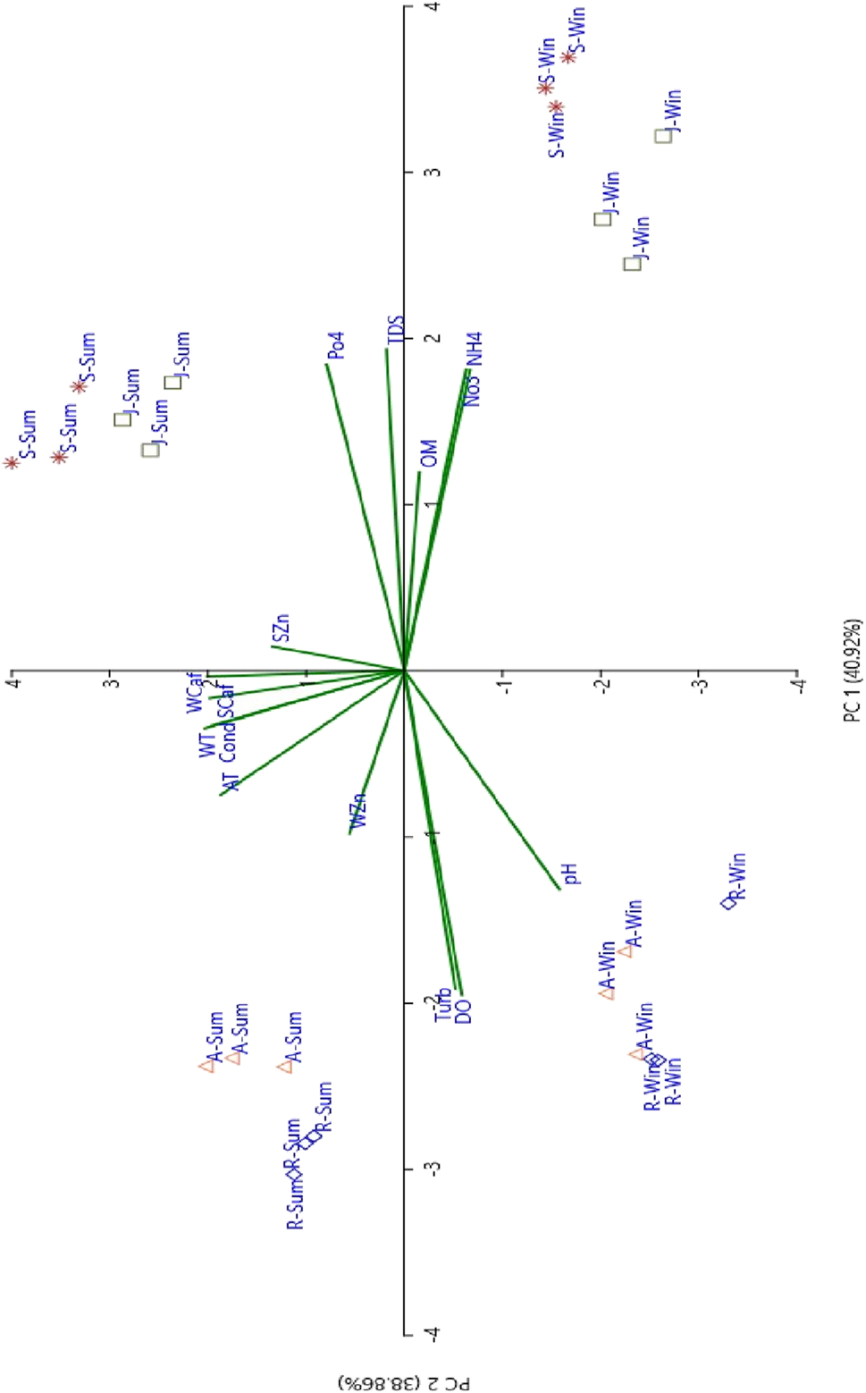
Principal component analysis (PCA) for the physicochemical parameters and caffeine and Zn concentrations in water and sediment at study sites during summer and winter seasons (after standardizing the collected data). Notation of variables: AT, air temperature (°C); WT, water temperature (°C); pH, water pH; Cond, conductivity (μS cm^−1^); TDS, total dissolved solids (ppm); Turb, turbidity (cm); DO, dissolved oxygen (mg L^−1^); OM, organic matter (%); PO_4_, phosphate (mg L^−1^); NO_3_, nitrate (mg L^−1^); NH_4_, ammonia (mg L^−1^); WCaf, water caffeine (μg L^−1^); SCaf, sediment caffeine (mg kg^−1^); WZn, water zinc (mg L^−1^); SZn, sediment zinc (mg kg^−1^)

Stepwise multiple regression was applied to select the most effective factors that have a significant impact on zinc and caffeine (Table 6). The results indicated that caffeine concentrations in water and sediment are correlated and mainly effected by temperature. WZn showed a negative correlation with NO_3_ while SZn had a positive correlation with SCaf and OM. The equations illustrating these relations are presented in Table 6. PCA revealed a direct correlation between WCaf and SCaf, WT, Cond, AT, WZn, Turb, DO and pH. However, there was a negative correlation between WCaf and SZn, PO_4_, TDS, OM, NH_4_ and NO_3_ (Fig. 6).

**Table 6:** Stepwise multiple regression between caffeine and Zn concentrations in water and sediment with the investigated physicochemical parameters

Dependent variable	Selected variable	*p* value	*R*^2^	Standard error of the estimate	Model *F* value (*p* value)	Regression equations
WCaf	Constant	0.001	0.89	6.878	54.43 (< 0.001)	WCaf = − 57.7 + 1.2 WT + 0.5 SCaf − 0.7 AT
	WT	< 0.001				
	SCaf	0.008				
	AT	0.01				
SCaf	Constant	0.058	0.84	0.218	55.14 (< 0.001)	SCaf = − 0.3 + 0.6 WCaf + 0.4 AT
	WCaf	0.001				
	WT	0.013				
WZn	Constant	< 0.001	0.19	0.058	5.26 (0.032)	WZn = 0.2 − 0.4 NO_3_
	NO_3_	0.032				
SZn	Constant	0.006	0.48	38.46	9.59 (0.001)	SZn = 46.9 + 0.6 SCaf + 0.4 OM
	SCaf	0.001				
	OM	0.014

## Discussion

Studies on the presence of caffeine in surface waters have been published around the world. There are only two publications on caffeine residues in the freshwater environment in Africa (Li et al. 2020a; Hawash et al. 2023). The present results demonstrated that caffeine concentrations in surface water samples from the Nile River ranged from 7.13 μg L^−1^ (R site during winter) to 40.32 μg L^−1^ (A site during summer). Abdallah et al. (2019) recorded that caffeine concentration in the surface water of the Nile River and El-Ebrahimiya Canal in Assiut City ranged between 7 and 54 ng L^−1^. This indicates that caffeine concentration increased over time. Also, the recorded concentrations are relatively higher than those recorded in other rivers and streams around the world. For example, caffeine was detected at quantities ranging from 0.006 to 0.250 g L^−1^ in Swiss lakes and rivers (Poiger et al. 2003). Caffeine concentrations ranged from 0.014 to 0.907 μg L^−1^ in samples collected upstream from Canadian Avon, Cornwallis and Annapolis rivers (Ghoshdastidar et al. 2015). Česen et al. (2019) reported that caffeine concentrations in Slovenian and Croatian Sava River ranged from 0.370 to 1.39 μg L^−1^. The caffeine concentrations in the urban rivers in China ranged from 0.066 to 8.571 μg L^−1^ (Zhou et al. 2016b). On the other hand, caffeine was observed in high levels in the Henares River, Spain (0.475 to 0.515 mg L^−1^) (Martínez Bueno et al. 2011); Lebanon rivers (10.2 mg L^−1^) (Mokh et al. 2017); the UK rivers (which reached 23.778 mg L^−1^) (Ebele et al. 2017); and Jundiaí River, Brazil (19.30 mg L^−1^) (de Sousa et al. 2014).

The present study demonstrated that caffeine concentrations of treated wastewater at the S site (effluents of Arab El-Madabegh WWTP, Assiut) ranged from 5.73 to 53.85 μg L^−1^. This range of concentration is higher than the concentration of caffeine recorded in 2019 for other effluents of WWTP in Assiut City which ranged between 70 and 1739 ng L^−1^ (Abdallah et al. 2019). A similar increasing caffeine concentration in effluent of WWTP over time was recorded for Canadian WWTP effluent. In 2006, caffeine was detected in Canadian WWTP effluent at concentrations ranging from 0.0017 to 1.244 μg L^−1^ (Hua et al. 2006); however, in 2015, it ranged from 0.013 to 115.141 μg L^−1^ (Ghoshdastidar et al. 2015). A relatively high concentration of caffeine was detected in WWTP effluent all over the world. For examples, caffeine concentrations in effluents of WWTPs reached 13 mg L^−1^ in Kuwait (Smith et al. 2015), 18 mg L^−1^ in Germany (Bahlmann et al. 2012), 28.8 mg L^−1^ in western Saudi Arabia (Alidina et al. 2014) and 34.2 mg L^−1^ in England (Baker and Kasprzyk-Hordern 2013). Mijangos et al. (2018) discovered the highest quantity of caffeine (66 mg L^−1^) in the effluent of a WWTP in Spain’s Urdaibai Estuary (Gernika).

The current results showed that caffeine concentrations in sediment reached 1.54 mg kg^−1^ at source site during summer. This indicated that caffeine was more concentrated in the sediment than in the water. According to Zhao et al. (2013), sediment functions as a sink, accumulating chemicals that may be discharged back into the aquatic environment. In fact, comparable analytical data from river sediment remains scarce. Martín et al. (2010) detected caffeine at concentrations of 7.21 μg kg^−1^ in sediment of the Guadiamar River in Seville, Southern Spain. Also, caffeine (659 ng/g) was detected in sediment from the Msunduzi River, South Africa (Matongo et al. 2015). On the other hand, caffeine pollution levels in coastal and marine ecosystems have been determined through sediment and water analysis (Nodler et al. 2014). Caffeine concentrations in coastal sediments have been documented in a few studies, with values ranging from 1.90 to 12.20 ng/g in Spain (Maranho et al. 2015) and from 0.31 to 23.4 ng/g in Brazil (Beretta et al. 2014).

The water Zn concentrations in the investigated sites ranged from 0.08 mg L^−1^ (S and J sites) to 0.22 mg L^−1^ (A site). Moreover, Zn concentration of sediments ranged between 28.59 and 155.02 mg kg^−1^ at J site in winter and summer seasons, respectively. The levels of Zn in studied samples often exceeded CCME (2007) guidelines for aquatic life. Samples collected from A site showed a relatively higher concentration than those from other sites which may be related to the effect of fertilizer plant emissions at Manqabad close to it. Mohamed et al. (2014) showed that the dust, fumes and gases that come out from fertilizer plants containing heavy metals affect the nearby area where soil samples collected near phosphate fertilizer production plants were found to be substantially enriched in heavy metals.

Examination of seasonal variations revealed that caffeine and Zn concentrations in water and sediments were higher during summer season at all studied sites. Seasonal variation in the concentrations of PPCPs (Ebele et al. 2020) and heavy metals (Hussein et al. 2006; Ololade et al. 2023) has been reported. It seems that the changes in human activities among different seasons might underlie the variation in caffeine and Zn existence from season to another. It is worth mentioning that Egyptian government uses the high dam to rationalize water during the winter season when crops do not need much irrigation water, also to implement many maintenance, disinfection and industrial works in the Nile River and its branches. According to Jagoda et al. (2015), greater caffeine residue concentrations were observed in Rudawa River water in Poland during the summer than in the fall, which was attributed to increased human agricultural activities and recreation in this area during the summer. Similarly, in Greece, the daylight hours increase throughout the summer, causing the caffeine content to rise to 9.48 mg L^−1^ in the summer compared to 1.93 mg L^−1^ in the winter (Kosma et al. 2014).

The maximal caffeine concentration was reported to be 44.6 mg L^−1^ in dry weather flow but 32.9 mg L^−1^ in wet weather flow in research done in Spain (Del Río et al. 2013). In addition, the median caffeine content in Guanajuato, Mexico, was 31.1 mg L^−1^ during the dry season, compared to 12.4 mg L^−1^ during the rainy season (Estrada-Arriaga et al. 2016). Caffeine residue levels in the influent of a WWTP in Greece peaked in winter at a mean concentration of 4.97 mg L^−1^, according to Papageorgiou et al. (2016), since caffeinated food and beverages were predominantly consumed by the public during this season. Caffeine concentration increased from 0.051 to 0.857 g L^−1^ in the Annapolis Royal STP effluent, while it decreased from 0.910 to 0.077 g L^−1^ in the Digby STP effluent in a study conducted in Canada (Ghoshdastidar et al. 2015), indicating possible seasonal variation in treatment efficiency or caffeine consumption, considering population change due to visitors. Ebele et al. (2020) showed that there is little information available on the variables influencing seasonal fluctuation of PPCPs in surface water and groundwater around the world. They illustrated that seasonal variations in concentrations of PPCPs could be related to numerous aspects distinctive to the investigated local environment, such as dilution by agricultural activities, rainfall and local community medicinal usage habits. It seems that the precise relationship between caffeine pollution levels and seasonal human inputs is currently unknown.

Concerning the physicochemical parameters, the present results indicate spatial and seasonal variations among investigated samples. These differences plus the variations in the amount of caffeine and zinc indicated that characteristics of water from S and J sites are significantly different from R and A sites. According to these differences, water samples from R and A sites during both study seasons cluster in one group while summer samples and winter samples from S and J sites were separated into two groups (Fig. 3). Overall, S and J sites have higher values of water temperature, Cond, Turb, OM, PO_4_, NO_3_, NH_4_, caffeine concentrations and Zn in sediment. In contrast, they have lower values of pH and DO. This indicated the effect of discharging of treated wastewater in the Nile River. Many previous studies indicated the effect of wastewater on environmental characteristics of surface water (Abdel-Satar et al. 2017; Berger et al. 2017; Onwona Kwakye et al. 2021; Rahman et al. 2021; Abdo et al. 2022; Tanjung et al. 2022). The microbial respiration as well as decomposing activities in wastewater consume part of the dissolved oxygen, affecting the pH value reflected in the changes of the other parameters like increasing of values of nutrients and NH_4_ (Onwona Kwakye et al. 2021; Abdo et al. 2022). Another factor that contributed to a low level of DO reality to the greater water temperature during the summer season (Table 1) was reduction due to the inverse connection between dissolved oxygen and temperature (Ice 2008). The relatively high concentrations of nitrates at all investigated sites may relate to runoff or leakage from artificial fertilizers, and water from a wastewater treatment plant, in addition to sludge, animal dung and septic tank waste (Zeidan 2017).

The current results show that water temperature, Cond and OM values still meet the quality standards for aquatic life at all study samples. On the contrary, WZn and NH_4_ concentrations have exceeded the quality standards in all samples. Likewise, samples from S and J sites having turbidity, DO and PO_4_ do not meet Egyptian standards for the water quality of the Nile River (EEAA 1999) in both study seasons. During summer season, S and J sites have pH values lower than the quality standards, while during winter, they have NO_3_ concentration higher than the quality standards. Only during summer, TDS has exceeded the standard for water of S site. These variations of physicochemical parameters led to that WQI of the collected samples ranged between good quality (water from R and A sites during summer) and marginal quality (water from S and J sites during summer and winter seasons). The relatively low value of WQI for the collected sample may relate to the high NH_4_ and Zn concentrations which related to the wastewater. Abdel-Satar et al. (2017) concluded that unceasing discharge of contaminants, generally heavy metals and nutrients, impacted the river health and reduced their self-purification capability, which affected the usability of Nile water for a variety of applications. The lowest WQI values were recorded in S (47.5%) and J (46.3%) sites during winter season. This is related to a drop in the Nile flow level in winter (as mentioned before), which tends to concentrate the ions. According to Abdelmageed et al. (2022), the Nile water level decreased by about 2.5 m in winter. They illustrated that increasing pollution caused by the Nile River water level declines has become Egypt’s primary issue, particularly with the building of the Grand Ethiopian Renaissance Dam (Abdel-Satar et al. 2017). On the other side, seasonal variations in natural processes such as temperature affect the quality of water in river and cause different features for altered seasons (Vega et al. 1998).

The current statistical analyses indicated that caffeine concentration in water and sediment, as well as Zn concentration in sediment, is positively correlated with WT and Cond and negatively with pH. Bethke et al. (2023) conducted a literature study on the impact of temperature, pH and combination stresses on pharmaceutical toxicity to organisms in aquatic ecosystems. They explained that the bioavailability of such micropollutants is tightly dependent on temperature and pH (Puckowski et al. 2016). According to Shiller and Boyle (1985), acidification and decreasing pH of river water may result in a great increase in dissolved Zn. In addition, temperature is also known to affect other factors such as pH, conductivity, dissolved gases and different alkalinity profiles (Beniwal et al. 2021). Berger et al. (2017) revealed that caffeine concentration is correlated with electrical conductivity as well as nitrite, nitrate and ammonium in German rivers. Rahman et al. (2021) observed that temperature is substantially correlated with pH in Bangladesh’s Turag River. They concluded that due to the wide variety of temperature tolerance in aquatic life, water temperature may not be as essential in pure water in contrast to polluted water, where temperature can have a significant impact on other factors.

The obtained results revealed that accumulation of Zn in sediment is highly related to organic matter. Similarly, Ololade et al. (2023) found that all examined elements including Zn are positively linked with sediment organic content in rivers in Southwestern Nigeria, whereas Hahn et al. (2018) demonstrated that organic matter in sediment has a significant impact on heavy metal absorption from the overlaying surface water. The present study showed a strong positive association between caffeine and Zn concentrations in sediments. This synergistic interaction poses an important environmental impact of heavy metals and pharmaceutical residues, as well as their bioavailability as a joint concern to the environment and human health. This emphasises the need of researching for such interactions in future research.

## Conclusion

The present results indicated that the discharge of treated wastewater in the Nile River is a significant source of caffeine and Zn in the environment. It seems that conventional WWTPs are incapable of removing caffeine and Zn completely. The caffeine and Zn residue levels in WWTP effluents peaked in the summer season, which showed a seasonal difference in treatment efficiency or caffeine consumption. The consumption of caffeine and Zn is expected to increase with the increase in the human population and activities. The variations in human social activities between seasons may impact anthropogenic inputs of caffeine and Zn in the environment, explaining the fluctuation in caffeine and Zn occurrence from season to season. However, the precise relationship between caffeine and Zn pollution levels and seasonal human inputs remains unclear. The decrease in Nile flow level during the winter season caused an increase in ion concentration in water, resulting in relatively lower WQI values for the collected samples in winter. The current results indicated that caffeine concentration in water and sediment is positively correlated with water temperature, conductivity and Zn concentration in sediment and negatively correlated with pH. As a result of the synergistic interaction between caffeine and Zn concentrations in the environment, great concerns should be raised about heavy metals and pharmaceutical residues having a significant environmental impact, which is a problem for ecological safety and human health. More attention should be paid to investigating such relationships in future research.

### Supplementary information

ESM 1 (DOCX 24 kb)

## Data Availability

The datasets generated during and/or analysed during the current study are available from the corresponding author on reasonable request.
