# Supplementary material for: Determination of caffeine in treated wastewater discharged in the Nile River with emphasis on the effect of zinc and physicochemical factors
Source: Environ Sci Pollut Res Int. 2024 Mar 26;31(19):28124–38. doi: 10.1007/s11356-024-32918-6 (PMC11058622; doi:10.1007/s11356-024-32918-6)
Supplement: Supplementary file 1 — (DOCX 24 kb) [file 11356_2024_32918_MOESM1_ESM.docx]

Supplementary Materials for

**Determination of caffeine in treated wastewater discharged in the Nile River with emphasis on the effect of Zinc and physicochemical factors**

Nouran A. I. Tawfik ^1^, Zienab A. El-Bakary^1^ and Khaleid F. Abd El-Wakeil^1*^

#### ^1^ Zoology and Entomology Department, Faculty of Science, Assiut University, Assiut, Egypt

*^*^ Corresponding author:* [kfwakeil@yahoo.com](mailto:kfwakeil@yahoo.com), [kfwakeil@aun.edu.eg](mailto:kfwakeil@aun.edu.eg)

SM1. Two-way ANOVA for Physicochemical parameters at the investigated sites during summer and winter seasons.

SM2. Two-way ANOVA for caffeine and Zn concentrations in water and sediment at the investigated sites during summer and winter seasons.
